# Drought Stress Memory at the Plant Cycle Level: A Review

**DOI:** 10.3390/plants10091873

**Published:** 2021-09-10

**Authors:** Cécile Jacques, Christophe Salon, Romain L. Barnard, Vanessa Vernoud, Marion Prudent

**Affiliations:** Agroécologie, AgroSup Dijon, INRAE, Université de Bourgogne, Université Bourgogne Franche-Comté, F-21000 Dijon, France; cecile.jacques@inrae.fr (C.J.); christophe.salon@inrae.fr (C.S.); romain.barnard@inrae.fr (R.L.B.); vanessa.vernoud@inrae.fr (V.V.)

**Keywords:** water stress, resilience, plant-microbe interplay, priming, memory genes, soil legacy

## Abstract

Plants are sessile organisms whose survival depends on their strategy to cope with dynamic, stressful conditions. It is urgent to improve the ability of crops to adapt to recurrent stresses in order to alleviate the negative impacts on their productivity. Although our knowledge of plant adaptation to drought has been extensively enhanced during the last decades, recent studies have tackled plant responses to recurrent stresses. The present review synthesizes the major findings from studies addressing plant responses to multiple drought events, and demonstrates the ability of plants to memorize drought stress. Stress memory is described as a priming effect allowing a different response to a reiterated stress when compared to a single stress event. Here, by specifically focusing on water stress memory at the plant cycle level, we describe the different underlying processes at the molecular, physiological and morphological levels in crops as well as in the model species *Arabidopsis thaliana*. Moreover, a conceptual analysis framework is proposed to study drought stress memory. Finally, the essential role of interactions between plants and soil microorganisms is emphasized during reiterated stresses because their plasticity can play a key role in supporting overall plant resilience.

## 1. Introduction

The world’s population should reach around 9.1 billion in 2050. An important increase in food demand is already being observed [1]. At the same time, agricultural production is facing the threat of climate change, which is characterized by more severe and frequent stressful conditions that hamper plant growth. Population increase and climate change are creating an unprecedent challenge in breeding plants that are more resilient to climate fluctuations in order to feed the world population. In this context, drought has been identified as the most important and harmful stress to plant production worldwide, affecting yield at several crucial moments during the crop cycle. While numerous studies have characterized the effects of various drought intensities occurring at different plant developmental stages [2,3], fewer have investigated the impact of recurrent drought periods on plant development and growth [4,5,6].

Plants are able to “remember” a stress event and to modify their behavior in response to a subsequent stress [6]. This so-called memory is defined as “an ability to access past experience so that new responses incorporate relevant information from the past”, and “information storage of previous signaling, with the ability to retrieve the information at a much later time.” [7].

Lämke and Bäurle [8] defined three different types of stress memory: (i) somatic stress memory, mitotically heritable that lasts only during the lifespan of an organism; (ii) intergenerational stress memory that is observable only in the first stress-free offspring generation and (iii) transgenerational memory that is meiotically heritable and observable after more than two stress-free offspring generations. Somatic stress memory allows plants that have experienced a stress event to benefit from stored information for days, weeks or months and to adapt their response when facing a further stress. For example, this mechanism has been well characterized in cold hardening [9]. Moreover, the information derived from a previous stress can be passed on from parents to offspring through intergenerational and transgenerational stress memory [10,11]. This aspect is not discussed in the present review which focuses only on memory within the plant life cycle.

Plant stress memory was first observed in the 1990s when researchers noted that some plants developed an acquired systemic resistance to further infections after being exposed to a pathogen attack [12,13,14]. Since then, it has been found that plant memory allows plants to respond faster or stronger to a subsequent stress and may provide enhanced protection when compared to naïve plants that have never encountered any stress. The first studies exploring the topic led to major advances in improving the understanding of priming on abiotic constraints by documenting physiological, proteomic, transcriptional and epigenetic modifications leading to a stress imprint crucial for plant memory establishment [6].

In the present review, we illustrate how these mechanisms are interconnected during recurrent drought events and can help the plant to be more resilient. Moreover, because plants strongly interact with the soil and its components, we consider interactions between the plant and soil micro-organisms as another possible piece of the puzzle leading to plant drought memory. Indeed, soil microbial community composition and activity, as well as soil physico-chemical properties, are shaped by soil legacy effects, and could influence plant responses to a subsequent stress [15,16].

## 2. Water Stress Memory: From the Plant Side

Water stress memory has been explored in different crop species with specific focuses ranging from molecular to physiological underlying processes (Table 1). Because water stress events are likely to occur more frequently with climate change, plants may mobilize water stress memory from early stages in their life cycle to minimize or alleviate the negative impact of subsequent stresses on growth and production. In some cases, plant priming resulted in a higher and faster response to a subsequent stress (Table 1). To date, the general understanding of the mechanism is the following. First, a stress imprint is established during the first stress event, which involves different physiological and molecular mechanisms such as the accumulation of stress-responsive osmoregulating metabolites or the synthesis of protective proteins [9]. Second, during the post-stress recovery period this stored information allows the plant to switch into a permissive state, that allows a faster or stronger response to a subsequent stress. This information storage implies (i) an accumulation of proteins in an inactive conformation [17,18] and of metabolites and phytohormones, and (ii) epigenetic modifications through DNA methylation, histone modification or chromatin remodeling [9,17,19,20,21]. Chromatine plasticity, whether meiotically inherited or not, has a crucial role both during immediate stress response and in long term adaptation [22,23]. Third, during the subsequent stress, the prior recruitment of these different compounds reduces the time for their synthesis in large amounts, thus allowing a faster response [24,25].

**Figure 1:**
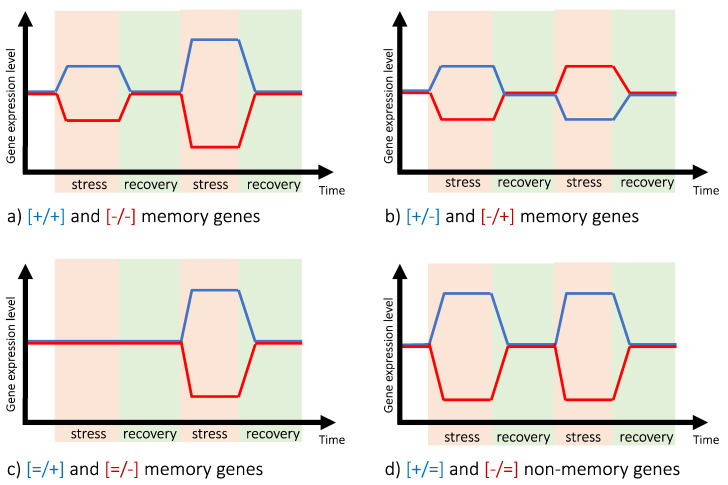
Different transcriptional patterns occurring in response to repeated stress treatments. Gene expression level is expressed relative to that of nonstressed plants. Memory genes have altered transcriptional responses to a subsequent stress. (**a**) Memory genes with [+/+] or [−/−] expression patterns; (**b**) Memory genes with [+/−] or [−/+] expression patterns; (**c**) Memory genes with [=/+] or [=/−] expression patterns (**d**) Non-memory genes responding similarly to each stress. Adapted from Ding et al. [26].

**Figure 2:**
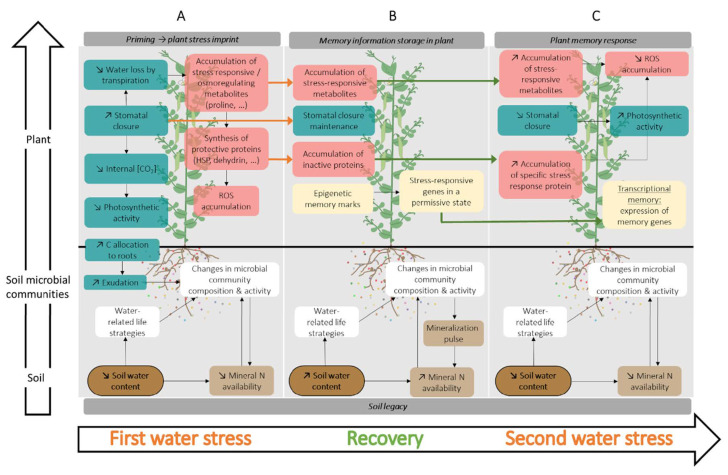
Water stress memory in the soil-plant system. (**A**) Effects of a first water stress on plant physiological (green boxes), biochemical (red boxes) and molecular (yellow boxes) plant responses, as well as on the soil microbial community (white and brown boxes). (**B**) Information storage by the plant and effects of rewatering on the soil. (**C**) Priming-enhanced plant response to a second water stress.

Pioneer work in *Arabidopsis thaliana* and *Zea mays* [25,26,27] showed that plants display a transcriptional stress memory in response to multiple exposures to drought, revealing the existence of memory genes. These genes are defined as producing different levels of transcripts in response to the first and the second stress, but basal levels of transcripts similar to that of the non-primed plants during the recovery period [25]. Following this concept, Figure 1 summarizes the classification of “memory genes” and “non-memory genes” into four categories according to the regulation of their expression during the second stress period, when compared to the first period. The expression of the [+/+] (or [−/−]) memory genes (Figure 1a) is induced (or repressed) during both the first and the second stresses when compared to the control, with priming increasing differential expression in the subsequent stress. Some memory genes can also display the opposite regulation in response to the first and second stresses. This is the case (i) for the [−/+] memory genes (Figure 1b), the expression of which is down-regulated during the first stress but up-regulated during the second stress, and (ii) for the [+/−] memory genes, the expression of which is up-regulated during the first stress but down-regulated during the subsequent stress. On the other hand, genes producing similar levels of transcripts in response to each stress are considered as “non-memory genes” and are annotated as [+/=] or [−/=] genes (Figure 1d) [26]. This succession of transcriptional events is translated into physiological changes [18], which are detailed below and summarized in Figure 2.

**Table 1:** Studies addressing recurrent water stresses in different crop species. For each study, the species, genotype and nature of each water stress is provided. FC, Field capacity; VWC, Volumetric Soil Water Content; SWC, Gravimetric Soil Water Content; DAS, Day After Sowing; DAP, Day After Planting.

***Plant Species*** (Genotype (s))	**PRIMING** **When?** How?	**SECOND STRESS** **When?** How?	**THIRD STRESS** **When?** How?	**Reference**
***Triticum aestivum*(Winter wheat)** (Luhan-7, Yuangmai-16)	**Tillering or jointing** Moderate drought (55–60% FC)	**Postanthesis** Severe drought (35–40% FC)		Abid et al., 2016 [28]
***Triticum aestivum*(Winter wheat)** (Luhan-7, Yuangmai-16)	**Tillering** Moderate drought (58–60% FC)	**Postanthesis** Severe drought (38–40% FC)		Abid et al., 2017 [29]
***Triticum aestivum*(Winter wheat)** (Yangmai-16)	**Seedling** Osmopriming (PolyEthylene Glycol, PEG)	**Tillering or jointing** Severe drought (35–40% FC)		Abid et al., 2018 [30]
***Oriza sativa*(Rice)** (BRS Querência, AN Cambara)	**Vegetative stage V5** 10% VWC vs. 40% VWC	**Preflowering stage R1-R2** 10% VWC vs. 40% VWC		Auler et al., 2017 [31]
***Oriza sativa*(Rice)** (BRS Querência, AN Cambara)	**Vegetative stage V5** 10% of pot capacity, during 7 days	**Preflowering stage R1-R2** 10% of pot capacity, during 7 days		Auler et al., 2021 [32]
***Solanum tuberosum*(Potato)** (JSY, CIP 706205)	**One-month old plant** Decrease of 10 to 20% of SWC	**One day after priming** Decrease of 10 to 20% of SWC		Chen et al., 2020 [33]
***Arabidopsis thaliana***	**Three-weeks old plant** Air drying: 2 h at 22 °C	**22 h after priming** Air drying: 2 h at 22 °C	**22 h after second stress** Air drying: 2 h at 22 °C	Ding et al., 2012 [25]
***Zea mays*(Mays)** (B73)	**Two-weeks old seedling** Air drying: 2 h at 22 °C	**22 h after priming** Air drying: 2 h at 22 °C		Ding et al., 2014 [26]
***Coffea canephora*(Cofee)** (clone 120 and 109)	**9 month old plants** 25% FC during 14 d	**≈ 10 days after priming** 25% FC, during 14 d	**≈ 10 days after second stress** 25% FC, during 14 d	Guedes et al., 2018 [34]
***Glycine max*(Soybean)** (Daepoong)	**7 days old plants** Water withholding during 4 days	**One day after priming** Water withholding during 4 days		Kim et al., 2020 [35]
***Arabidopsisthaliana*** (Transgenic recombinant aequorin)	**Seedling** Hydrogen peroxide Manitol	**6 to 7 days-old seedling** Hydrogen peroxide Manitol		Knight et al., 1998 [36]
***Beta vulgaris*(Sugar beet)** (Pauletta OVK, 8GK)	**35–54 DAS** Water withholding	**86–102 DAS** Water withholding	**135–151 DAS** Water withholding	Leufen et al., 2016 [37]
***Oriza sativa*(Rice)** (Zhonghua 11)	**4-weeks old seedling** Air drying: 80 min at 28 °C	**22 h after priming** Air drying: 80 min at 28 °C	**22 h after second** Air drying: 80 min at 28 °C	Li et al., 2019 [38]
***Arabidopsisthaliana*** (Wt, cfl mutant)	**Three-weeks old plant** Air drying: 90 min at 22 °C	**22 h after priming** Air drying: 90 min at 22 °C	**22 h after second stress** Air drying: 90 min at 22 °C	Liu et al., 2014 [39]
***Saccharum* spp.(Sugarcane)** (IACSP94-2094)	**55–days old plant** PEG for 5 days	**3 days after priming** PEG for 5 days	**3 days after second** PEG, during 5 days	Marcos et al., 2018a [40]
***Saccharum* spp.(Sugarcane)** (IACSP94-2094)	**6-month old plant** 20% vs. 60% VWC, during 9 days	**6 days after priming** 20% vs. 60% VWC, during 9 days	**6 days after second** 20% vs. 60% VWC, during 9 days	Marcos et al., 2018b [41]
***Solanum tuberosum*(Potato)** (Unica, Sarnav, Désirée)	**After tuber initiation or Seed tuber from previous experiment** 50% FC	**55 DAP** 50% FC		Ramirez et al., 2015 [42]
***Arabidopsisthaliana*** (Col-0)	**4 leaves stage** 5 mM NaCl	**10 days after priming** Water withholding for 2 weeks		Sani et al., 2013 [11]
***Arabidopsisthaliana*** (Col-0, *abf*, *snrk* and *aba* mutants)	**3-weeks old plant** Air drying: 90 to 120 min at 22 °C	**22 h after priming** Air drying: 90 to 120 min at 22 °C		Virlouvet et Fromm, 2015 [43]
***Zea mays*(Mays)** (cultivar B73)	**2-weeks old seedling** Air drying: 90 min at 22 °C	**22 h after priming** Air drying: 90 min at 22 °C		Virlouvet et al., 2018 [18]
***Triticum aestivum*(Spring wheat)** (Vinjett)	**Stem elongation (37 DAS)** or **Seedling (27 DAS) and Stem elongation (37 DAS)** Water withholding for 8 days	**15 days after anthesis** Water withholding for 8 days		Wang et al., 2014 [44]
***Oriza sativa*(Rice)** (Zhonghua 11)	**4-weeks old plant** Air drying: 80 min at 28 °C	**22 h after priming** Air drying: 80 min at 28 °C	**22 h after second** Air drying: 80 min at 28 °C	Yang et al., 2020 [45]

### 2.1. Photosynthesis and Energy-Related Mechanisms

Changes in photosynthesis mechanisms and energy balance have been highlighted in several studies addressing water stress memory. The responses differ based on whether the plants have vegetative storage organs (e.g., *Beta vulgaris*) or not, which implies that depending on the sink-source relationships during stress, physiological and biochemical plant parameters could be impacted differently [37]. In *Triticum aestivum*, plant priming (i.e., exposure to a first stress) increased chlorophyll and ribulose 1,5 bisphosphate carboxylase content as well as photosynthetic efficiency during a second stress. Thus, priming induces a higher maintenance of photosynthetic apparatus during the subsequent stress [28,29,30]. These contrasted physiological responses following priming can be related to different molecular responses via transcriptional memory [18,25,26,43]. Indeed, in *Zea mays* and *Arabidopsis thaliana*, among the 556 memory genes common to the two species, 18% were related to photosynthetic activity and energy balance. In maize, [=/−] and [=/+] memory genes (Figure 1c) encode proteins involved in light harvesting, energy transport, non-photochemical quenching and overall photosynthesis, including enzymes of the Calvin-Benson-Bassham cycle [18]. In addition, the down-regulation of a memory gene encoding a chloroplastic ATP synthase during the second stress suggests the role of a transcriptional component in altering energy-dependent quenching sensitivity, ultimately leading to the protection of the photosynthetic apparatus from drought [18]. Similar results were observed in *Glycine max*, for which some drought-repressed memory genes were also related to photosynthesis activity [35].

Depending on crop biomass partitioning to the different organs, physiological and biochemical plant parameters may be impacted differently during stress [37]. In *Beta vulgaris* exposed to three water stress events, all stresses reduced plant chlorophyll content, but the magnitude of the effect was lower during the second stress and even lower during the third stress [37]. Meanwhile, all three stresses reduced net photosynthesis and transpiration to the same extent. Thus, although this process cannot be generalized to all situations, a first water stress can improve plant response to a subsequent stress by dampening the impact of the second stress on plant photosynthesis and energy mechanisms, thereby sustaining a better carbon status.

### 2.2. Osmotic Adjustment and Plant Water Status

Under water deficit, plant ABA synthesis induces stomatal closure through the regulation of Ca^2+^ in the guard cells, preventing water loss. The regulation of stomatal aperture in guard cells is also largely dependent on the expression of members of the SnRK2 gene family that mediate both ABA-dependent and independent responses [43,46]. In parallel, the accumulation of solutes frequently involved during water stress, such as proline [47], compensates the drop in water potential associated with decreased water content in plant tissue.

Osmotic adjustment for water status maintenance is involved in water stress plant memory. In primed Arabidopsis thaliana plants, an increase in the magnitude of the cytosolic free Ca^2+^ response to subsequent osmotic stress has been observed and could be involved in better tolerance to subsequent abiotic stress [36]. Virvoulet and Fromm [43] showed that both physiological and transcriptional memories occurred in *Arabidopsis thaliana* guard cells in response to repeated dehydration stresses. Moreover, transcriptome analyses upon repetitive stress exposures in *Zea mays* revealed that a large proportion of the [−/−] and [+/+] memory genes encoded proteins with membrane-associated functions such as dehydrins [+/+], transmembrane transporters for inorganic phosphate and sucrose [−/−], and regulators of water and potassium uptake and transport [26]. Similarly, enzymes involved in osmolyte synthesis and proline biosynthesis were encoded by [+/+], [−/+] and [+/−] memory genes in both *Arabidopsis thaliana* and *Zea mays* [25,26]. 

In primed *Oriza sativa* plants, proline accumulation was increased compared to naïve plants [31], which could contribute to the enhancement of leaf water potential and the maintenance of plant water status during the subsequent drought.

### 2.3. Cellular Protective Functions: Detoxifying Systems and Chaperones

Protective and detoxifying functions are crucial for plant stress memory because they minimize the impact of drought-induced oxidative stress by maintaining cellular metabolism. Studies by Abid and collaborators [28,29,30] showed in *Triticum aestivum* plants that priming enhanced photoprotection during the second stress via a better detoxifying system. This included lower reactive oxygen species (ROS) accumulation and lipid peroxidation, and a higher activity of antioxidant enzymes such as catalase, ascorbate peroxidase, glutathione reductase and superoxide dismutase.

Moreover, in primed *Silene dioica* plants, chlorophyll a/b ratio is higher after a repeated stress than after a single stress, suggesting a decrease in ROS production and photo-oxidative stress if a subsequent stress occurs [48]. In *Arabidopsis thaliana*, *Zea mays* and *Glycine max*, [+/+] memory genes encoded proteins related to protective functions (dehydrins, HSP, chaperones implicated in protein folding) and metabolic enzymes for the synthesis of protective molecules (i.e., osmolytes) [25,26]. 

Plant water stress memory thus involves improving the detoxifying system thanks to enhanced antioxidant enzyme activities and better protective functions via chaperone proteins, allowing plants to improve their responses to oxidative stress and to sustain protein activity.

### 2.4. Epigenetic and Molecular Mechanisms Involved in Transcriptional Memory Establishment

Epigenetic modifications related to a given stress modulate gene expression during a subsequent stress. They can contribute to transcriptional memory through memory genes, non-memory genes and transcription factors. 

Two distinct marks have been characterized on memory genes during recovery periods that followed dehydration stress periods in *Arabidopsis thaliana* [25]. These memory marks include histone modifications, such as the maintenance of a high level of trimethylated histone H3Lys4 nucleosomes (H3K4me3) and stalled Ser5P RNA Polymerase II (Ser5P pol II) at stress memory genes during recovery, even though their transcription level was low during recovery. These epigenetic marks play a role in transcriptional memory, since they are enriched during stress periods and maintained at a certain level during recovery periods [5]. The accumulation of H3K4me3 is not specific to drought memory, as it has also been observed in heat stress memory [49] and salinity [11]. In contrast, elevated levels of Ser5P pol II have been poorly described in plants but were shown to be prevalent in genes involved in development and response to stimuli in animals [50]. The factors or genes that cause ser5P pol II and H3K4me3 association with memory genes and transcriptional stress memory are still unknown. The histone H3K4 methyltransferase ATX1 (TRITHORAX-LIKE 1) is necessary but not sufficient, as the transcriptional memory response in the atx1 mutant is attenuated but not eliminated. Similarly, the involvement of ABA and ABA-regulated transcription factors such as AREB1, AREB2 (ABSCISIC ACID–RESPONSIVE ELEMENT BINDING PROTEIN 1 and 2, respectively), and ABF3 (ABSCISIC ACID RESPONSIVE ELEMENTS-BINDING FACTOR 3) are important for the magnitude of induction of some memory genes, but not essential for the memory response to occur [25]. More recently, the potential implication of DNA-methylation in drought stress-memory was demonstrated in the resurrection plant *Boea hygrometrica* [51]. Up-regulation of memory genes including pre-mRNA-splicing factor 38A, vacuolar amino acid transporter 1-like, and UDP-sugar pyrophosphorylase, was associated with promoter methylation variations in the CG and CHG contexts. Although these epigenetic modifications are generally not meiotically inherited, they can, in some cases, be passed on to the next generation and form a transgenerational memory. This mechanism could be of great interest for breeding purposes, especially towards the improvement of long-term plant adaptation to fluctuating environments [10,52]. 

Non-memory genes are involved in transcriptional memory by playing a role in the implementation of epigenetic marks on memory genes. Non-memory genes have been identified, such as a [−/=] putative methyltransferase with a DNA-binding domain and a [+/=] gene annotated “nucleosome remodeling factor” [34]. 

The activity of transcription factors (TF) is involved in stress memory, although the transcriptional memory pattern of a TF does not necessary determine the memory pattern of its targets (even if direct). For example, the memory expression pattern of the bHLH MYC2 transcription factor under repeated dehydration stresses did not correlate with the non-memory expression pattern of its target gene RD22 [27]. Both in *Zea mays* and *Arabidopsis thaliana*, about 10% of the drought stress memory genes encoded TFs, but some families were identified as species-specific. For instance, the NAC (NAM, ATAF and CUC) family TFs with a [+/+] signature and the integrase-type AP2/ERF (APETALA 2/ERE binding factor) family members with a [+/−] signature were highly represented in maize, while TFs from the AP2/ERF, bHLH (basic helix-loop-helix) and ZF (Zinc finger) families were more specific to *Arabidopsis thaliana* [26].

One additional level in memory gene regulation could involve small RNAs, in particular microRNA (miRNA), as shown for heat stress (HS) memory. In *Arabidopsis thaliana*, thermotolerance is compromised in miRNA pathway mutants such as ago1 (argonaute1) and dcl1 (dicer-like1) [53]. Functional analysis demonstrated that mir156 is specifically required for HS memory through the repression of its targets SPL2 (SQUAMOSA promoter binding protein-like 2) and SPL11. In addition, mir156 over-expression enhanced and prolonged the HS memory effect [53]. In *Medicago sativa* (alfalfa) mir156 over-expressor lines showed improved drought stress tolerance [54], and drought-responsive miRNAs have been identified in numerous crop species including legumes [55], cereals ([56] for a review) and *Solanum lycopersicum* [57]. To what extent miRNA could mediate drought stress memory remains to be elucidated. 

### 2.5. Plant Biomass and Productivity

Plant biomass production and yield are the main macroscopic indicators of drought stress memory, as they integrate the different molecular mechanisms and physiological processes involved in plant response to repeated stresses. For instance, primed *Triticum aestivum* plants at the tillering stage produced higher yields than non-primed plants after subsequent stresses, likely through modulations of growth hormone levels (i.e., higher cytokinin, indole-3-acetic acid, gibberellin contents, and lower ABA content in the primed plants) [29]. Priming at the seed stage can have long-lasting effects. Osmopriming *Triticum aestivum* seeds with polyethylene glycol (PEG) before the occurrence of drought at tillering and jointing stages led to sustained relative growth rate during stress and higher final grain yield production [30]. 

However, drought stress memory cannot be generalized, since its establishment is species and genotype-dependent. The transcriptional memory differences that exist between *Arabidopsis thaliana* and *Zea mays* subjected to repeated dehydration stresses (described above) may reflect differences in photosynthetic and related metabolism functions between C3 and C4 plants [26], while we hypothesize that differences in physiological memory between crops allocating more of their resources to non-reproductive organs (e.g., tuber) versus those remobilizing resources towards seeds could be explained by contrasted source-sink relationships. Differences in drought stress memory abilities between genotypes have been highlighted in several species. In *Solanum tuberosum*, although priming induced an increase of tuber amino acid content in the two varieties Sarnav and Unica, priming only resulted in a higher tuber yield for the Sarnav variety [42]. In *Triticum aestivum*, the higher tolerance of the Luhan cultivar than Yuangmai cultivar has been related to a higher stress memory ability in response to recurrent droughts [28].

### 2.6. Trade-off between Stress Memory and Stress Forgetfulness (Memory Resetting)

Plant priming induced by a preexposure to a first stress can allow a faster or a more intense response to a subsequent stress [24], and its cost is estimated to be relatively low compared to naïve plants which constitutively express stress-related genes [6,9,58,59]. Yet, sustaining a primed state via short-term (morphological acclimation, physiological changes, molecular and metabolic alterations) and long-term mechanisms (epigenetic processes) is energy-consuming and can negatively impact other biological processes such as plant growth (including photosynthesis and resource allocation) or development [22]. As such, it can be advantageous to learn to forget. Resetting (aka stress forgetfulness) has been proposed as the main plant strategy to fine-tune growth in fluctuating and unpredictable environmental conditions [22]. Throughout the plant cycle, alternating periods of establishing memory and resetting it could be driven by RNA metabolism, post-transcriptional gene silencing or RNA-directed DNA methylation [22]. To our knowledge, the resetting of a plant primed state after a drought event has not been studied yet. However, in the case of heat stress memory, resetting involves autophagy-mediated degradation of HSP proteins during recovery in order to reset the cell proteome [60]. Thus, stress memory establishment and its resetting appear to be coordinated by fine-tuned mechanisms that modulate memory duration (e.g., four days in a study of heat stress memory) [60]. As a result, this process contributes to alleviate the negative impact of recurrent stresses on plant biomass production [9]. Too little tangible evidence is currently available to understand the dynamics and the regulation of the trade-off between stress memory and stress resetting, thus opening a huge science front for the coming years.

**Figure 3:**
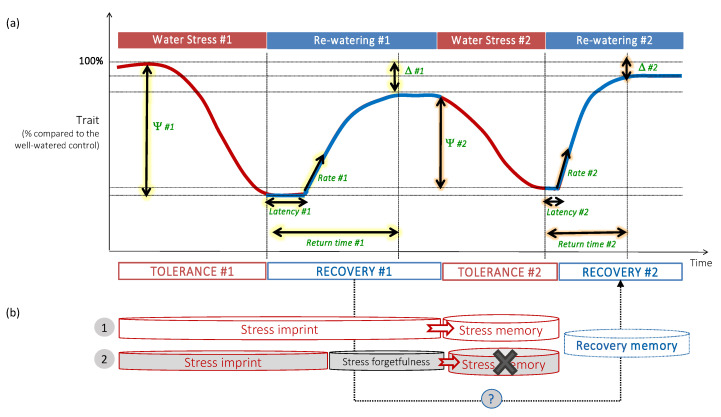
Conceptual framework of plant water stress memory and putative recovery memory. (**a**) A first stress (*#1*) induces the decrease of a value of a trait with a given intensity (Ψ *#1*) and its recovery during rewatering until plateauing. During a subsequent water stress (*#2*), the intensity decrease (Ψ *#2*) may be less important than during the first stress. During each re-watering period, the recovery capacity may be characterized by different variables (in green): recovery initiation latency, recovery rate, return time to plateau, and delta (Δ, the difference in trait value at the plateau between well-watered plants and those under water stress). During the second recovery, the trait value increase may be closer to the control value (Δ *#2*) than after the first recovery (Δ *#1*), implying a putative recovery memory process. (**b**) A stress memory can be established when the stress imprint is stored until the beginning of the second water deficit period (case 1). A stress memory cannot occur when the stress imprint is followed by memory resetting (stress forgetfulness) such as in case 2.

To that end, we propose a conceptual analysis framework (Figure 3) including stress memory, stress forgetfulness and recovery memory that could help in understanding the dynamics of these processes. This conceptual framework follows the general concept initially developed by Couchoud et al. [61], which involves the characterization of different parameters during both water stress and recovery periods: impact of the first stress, dynamics of the recovery after each stress, and duration of stress memory. Finally, a better characterization of the regulatory dynamics underlying stress memory would make it possible to predict plant responses to multiple stresses based on process-based modelling. Moreover, thanks to technical advances and the increased availability of more powerful tools and methods, a deeper understanding of the dynamics of stress memory/forgetfulness will be possible. These tools include high-throughput phenotyping of shoots and roots that allow screening a large number of genotypes under controlled conditions (major plant phenotyping centers are part of the International Plant Phenotyping Network; www.plant-phenotyping.org, accessed on 7 September 2021). Molecular tools and methods based on multiomics and systems biology approaches can help in identifying the main regulators that are necessary for genetic improvement [62]. Recent advances in epigenetics allowed the construction of “epi-populations” that can reveal “epi-alleles” whose variants can also be considered as breeding targets [10]. Then, once the candidate genes have been identified, genome editing tools such as CRISPR-Cas9 [63] will be useful for gene functional validation. Finally, speed-breeding techniques, by shortening the growth cycle of plants, would greatly accelerate the genetic improvement of crops [64].

## 3. Water Stress Memory and the Plant x Microbiome Interplay

Soil microorganisms establish strong interactions with plants in relation to nutrient acquisition, protection against pathogens and beneficial physico-chemical changes in the soil. Soil moisture is a key driver of microbial community composition and activity. Due to the much more rapid turnover of soil microorganisms compared to plants, individual-level memory is less relevant for soil microbes at the community scale. Instead, the term “legacy” is typically used for describing the effects of changes in environmental conditions on soil microbial community over time [65]. 

Microbial adaptation to drought periods followed by rewetting involves different coping mechanisms, such as sporulation or the production of osmolytes to resist osmotic stress, which are related to life-strategies along a drought-resistant to opportunistic gradient [66]. In addition to the inherent large and rapid change in soil water potential, rewetting triggers C and N mineralization bursts that constitute an additional modification in soil environmental conditions [67,68]. Advances in molecular microbiology over the last decades, based on next-generation sequencing and including metagenomics and metatranscriptomics, have shed light on the dynamic responses of soil microorganisms and their activity to dry-wet cycles. Exposing a microbial community to drought can improve its resistance to subsequent drought and rewetting events [69,70]. More generally, legacy effects of past drought conditions can shape the response of soil microbial communities to future droughts [65,70,71]. As a consequence, microbial legacy can affect major soil functions such as decomposition [72] or decomposition-related mechanisms [73,74], which will directly influence the plant’s nutritional status and likely affect ecosystem properties. For example, the effects of an anomalously warm year can contribute to changes in ecosystem functioning which are related to plant-microbial interactions, and can persist several years after a drought event [75].

Do stress legacy effects of drought on microorganisms modify the response of plants to a subsequent stress? Microbial communities that are adapted to water stress can improve plant fitness and resistance to drought [76,77,78]. Similarly, microbial communities subjected to previous drought conditions can alter the direction of plant-soil feedback [79]. Conversely, do plant memory effects shape the response of soil microorganisms to a subsequent drought? Increased rhizodeposition under moderate drought stress is a generally observed trend, despite species-specific variability, which is expected to directly stimulate microbial community functioning [80]. Under suboptimal conditions, plants can select beneficial microorganisms in their rhizosphere through the exudation of different compounds that are available for soil microorganisms [81]. For instance, root secretions of hormones involved in plant immune responses, such as salicylic acid and jasmonic acid, can shape rhizosphere and root microbiome assembly and functionality [77]. The enrichment of soil with plant-protective microorganisms can be beneficial for the plant during its cycle, but also for further plant generations growing in the same soil [82]. 

The soil legacy effect could thus be a key driver of terrestrial plant community composition and productivity, with effects that persist over time [76]. We suggest they should explicitly be taken into account when addressing an extended framework of plant stress memory. The challenge, however, is that microbial legacy takes place over longer periods of time than plant memory effects, since time is required for changes in composition and activity to become established in a fundamentally dynamic community.

## 4. Conclusions

Plant water stress memory involves processes associated with photosynthesis, energy mechanisms, osmotic adjustment, cellular protective functions, and water status maintenance. Memory mechanisms are best known at the shoot level, yet it is essential to characterize those at the root level. Indeed, the role of the root system in water and nutrient uptake is crucial for plant growth, development and yield. From a wider point of view, the close interaction between root system memory and the microbiome legacy in the soil represents the next challenge to tackle. Most studies on plant memory have been conducted on cereal or vegetative storage crops, while legume stress memory has scarcely been addressed. However, legumes are a model of choice for understanding the memory of plants in interaction with soil microorganisms, due to their ability to establish symbiotic relationships with rhizobium and mycorrhizal fungi. A more holistic and dynamic approach of plant resilience would i) bring plant-microbial interactions into the picture and ii) improve the understanding of recovery memory after a stress period and the fine trade-off between plant memory and forgetfulness. Increased knowledge on plant resilience, that includes stress memory, thus appears to open up new perspectives in the general context of food security under a changing climate.

## References

[1] Food and Agriculture Organization of the United Nations (2009). Livestock in the Balance. The state of Food and Agriculture 2009.

[2] Farooq M., Gogoi N., Barthakur S., Baroowa B., Bharadwaj N., Alghamdi S.S., Siddique K.H.M. (2017). Drought stress in grain legumes during reproduction and grain filling. J. Agron. Crop. Sci..

[3] Anjum S.A., Ashraf U., Zohaib A., Tanveer M., Naeem M., Ali I., Tabassum T., Nazir U. (2017). Growth and developmental responses of crop plants under drought stress: A review. Zemdirb. Agric..

[4] Li X., Liu F. (2016). Drought stress memory and drought stress tolerance in plants: Biochemical and molecular basis. Drought Stress Toler. Plants.

[5] Avramova Z. (2015). Transcriptional ‘memory’ of a stress: Transient chromatin and memory (epigenetic) marks at stress-response genes. Plant J..

[6] Bruce T.J.A., Matthes M.C., Napier J.A., Pickett J.A. (2007). Stressful “memories” of plants: Evidence and possible mechanisms. Plant Sci..

[7] Trewavas A. (2003). Aspects of plant intelligence. Ann. Bot..

[8] Lämke J., Bäurle I. (2017). Epigenetic and chromatin-based mechanisms in environmental stress adaptation and stress memory in plants. Genome Biol..

[9] Hilker M., Schmülling T. (2019). Stress priming, memory, and signalling in plants: Stress priming, memory, and signalling in plants. Plant Cell Environ..

[10] Mladenov V., Fotopoulos V., Kaiserli E., Karalija E., Maury S., Baranek M., Segal N., Testillano P., Vassileva V., Pinto G. (2021). Deciphering the epigenetic alphabet involved in transgenerational stress memory in crops. Int. J. Mol. Sci..

[11] Sani E., Herzyk P., Perrella G., Colot V., Amtmann A. (2013). Hyperosmotic priming of arabidopsis seedlings establishes a long-term somatic memory accompanied by specific changes of the epigenome. Genome Biol..

[12] Metraux J.P., Signer H., Ryals J., Ward E., Wyss-Benz M., Gaudin J., Raschdorf K., Schmid E., Blum W., Inverardi B. (1990). Increase in salicylic acid at the onset of systemic acquired resistance in cucumber. Science.

[13] Malamy J.E. (2005). Intrinsic and environmental response pathways that regulate root system architecture. Plant Cell Environ..

[14] Ward E., Uknes S., Dincher S., Wiederhold L., Alexandrer C., Ahl-Goy P., Metraux P., Ryals A. (1991). Coordinate gene activity in response to agents that induce systemic acquired resistance. Plant Cell.

[15] Bengtsson J., Angelstam P., Elmqvist T., Emanuelsson U., Folke C., Ihse M., Moberg F., Nyström M. (2003). Reserves, resilience and dynamic landscapes. J. Hum. Environ..

[16] Walter J., Jentsch A., Beierkuhnlein C., Kreyling J. (2013). Ecological stress memory and cross stress tolerance in plants in the face of climate extremes. Environ. Exp. Bot..

[17] Hilker M., Schwachtje J., Baier M., Balazadeh S., Bäurle I., Geiselhardt S., Hincha D.K., Kunze R., Mueller-Roeber B., Rillig M.C. (2016). Priming and memory of stress responses in organisms lacking a nervous system: Priming and memory of stress responses. Biol. Rev..

[18] Virlouvet L., Avenson T.J., Du Q., Zhang C., Liu N., Fromm M., Avramova Z., Russo S.E. (2018). Dehydration stress memory: Gene networks linked to physiological responses during repeated stresses of *Zea mays*. Front. Plant Sci..

[19] Kinoshita T., Seki M. (2014). Epigenetic memory for stress response and adaptation in plants. Plant Cell Physiol..

[20] Martienssen R.A. (2001). DNA methylation and epigenetic inheritance in plants and filamentous fungi. Science.

[21] Sahu P.P., Pandey G., Sharma N., Puranik S., Muthamilarasan M., Prasad M. (2013). Epigenetic mechanisms of plant stress responses and adaptation. Plant Cell Rep..

[22] Crisp P.A., Ganguly D., Eichten S.R., Borevitz J.O., Pogson B.J. (2016). Reconsidering plant memory: Intersections between stress recovery, RNA turnover, and epigenetics. Sci. Adv..

[23] Mirouze M., Paszkowski J. (2011). Epigenetic contribution to stress adaptation in plants. Curr. Opin. Plant Biol..

[24] Walter J., Nagy L., Hein R., Rascher U., Beierkuhnlein C., Willner E., Jentsch A. (2011). Do plants remember drought? Hints towards a drought-memory in grasses. Environ. Exp. Bot..

[25] Ding Y., Fromm M., Avramova Z. (2012). Multiple Exposures to drought “train” transcriptional responses in arabidopsis. Nat. Commun..

[26] Ding Y., Virlouvet L., Liu N., Riethoven J.-J., Fromm M., Avramova Z. (2014). Dehydration stress memory genes of *Zea mays*; Comparison with *Arabidopsis thaliana*. BMC Plant Biol..

[27] Ding Y., Liu N., Virlouvet L., Riethoven J.-J., Fromm M., Avramova Z. (2013). Four distinct types of dehydration stress memory genes in *Arabidopsis thaliana*. BMC Plant Biol..

[28] Abid M., Tian Z., Ata-Ul-Karim S.T., Liu Y., Cui Y., Zahoor R., Jiang D., Dai T. (2016). Improved tolerance to post-anthesis drought stress by pre-drought priming at vegetative stages in drought-tolerant and -sensitive wheat cultivars. Plant Physiol. Biochem..

[29] Abid M., Shao Y., Liu S., Wang F., Gao J., Jiang D., Tian Z., Dai T. (2017). Pre-drought priming sustains grain development under post-anthesis drought stress by regulating the growth hormones in winter wheat (*Triticum aestivum* L.). Planta.

[30] Abid M., Hakeem A., Shao Y., Liu Y., Zahoor R., Fan Y., Suyu J., Ata-Ul-Karim S.T., Tian Z., Jiang D. (2018). Seed osmopriming invokes stress memory against post-germinative drought stress in wheat (*Triticum aestivum* L.). Environ. Exp. Bot..

[31] Auler P.A., do Amaral M.N., dos Santos Rodrigues G., Benitez L.C., da Maia L.C., Souza G.M., Braga E.J.B. (2017). Molecular responses to recurrent drought in two contrasting rice genotypes. Planta.

[32] Auler P.A., Souza G.M., da Silva Engela M.R.G., do Amaral M.N., Rossatto T., da Silva M.G.Z., Furlan C.M., Maserti B., Braga E.J.B. (2021). Stress memory of physiological, biochemical and metabolomic responses in two different rice genotypes under drought stress: The scale matters. Plant Sci..

[33] Chen Y., Li C., Yi J., Yang Y., Lei C., Gong M. (2019). Transcriptome response to drought, rehydration and re-dehydration in potato. Int. J. Mol. Sci..

[34] De Guedes F.A.F., Nobres P., Rodrigues Ferreira D.C., Menezes-Silva P.E., Ribeiro-Alves M., Correa R.L., DaMatta F.M., Alves-Ferreira M. (2018). Transcriptional memory contributes to drought tolerance in coffee (*Coffea canephora*) Plants. Environ. Exp. Bot..

[35] Kim Y.-K., Chae S., Oh N.-I., Nguyen N.H., Cheong J.-J. (2020). Recurrent drought conditions enhance the induction of drought stress memory genes in *Glycine max* L.. Front. Genet..

[36] Knight H., Brandt S., Knight M.R. (1998). A history of stress alters drought calcium signalling pathways in arabidopsis: Stress history in arabidopsis. Plant J..

[37] Leufen G., Noga G., Hunsche M. (2016). Drought stress memory in sugar beet: Mismatch between biochemical and physiological parameters. J. Plant Growth Regul..

[38] Li P., Yang H., Wang L., Liu H., Huo H., Zhang C., Liu A., Zhu A., Hu J., Lin Y. (2019). Physiological and transcriptome analyses reveal short-term responses and formation of memory under drought stress in rice. Front. Genet..

[39] Liu N., Fromm M., Avramova Z. (2014). H3K27me3 and H3K4me3 chromatin environment at super-induced dehydration stress memory genes of *Arabidopsis thaliana*. Mol. Plant.

[40] Marcos F.C.C., Silveira N.M., Mokochinski J.B., Sawaya A.C.H.F., Marchiori P.E.R., Machado E.C., Souza G.M., Landell M.G.A., Ribeiro R.V. (2018). Drought tolerance of sugarcane is improved by previous exposure to water deficit. J. Plant Physiol..

[41] Marcos F.C.C., Silveira N.M., Marchiori P.E.R., Machado E.C., Souza G.M., Landell M.G.A., Ribeiro R.V. (2018). Drought tolerance of sugarcane propagules is improved when origin material faces water deficit. PLoS ONE.

[42] Ramírez D.A., Rolando J.L., Yactayo W., Monneveux P., Mares V., Quiroz R. (2015). Improving potato drought tolerance through the induction of long-term water stress memory. Plant Sci..

[43] Virlouvet L., Fromm M. (2015). Physiological and transcriptional memory in guard cells during repetitive dehydration stress. New Phytol..

[44] Wang X., Vignjevic M., Jiang D., Jacobsen S., Wollenweber B. (2014). Improved tolerance to drought stress after anthesis due to priming before anthesis in wheat (*Triticum aestivum* L.) Var. Vinjett. J. Exp. Bot..

[45] Yang H., Li P., Jin G., Gui D., Liu L., Zhang C. (2020). Temporal regulation of alternative splicing events in rice memory under drought stress. Plant Divers..

[46] Fujii H., Zhu J.-K. (2009). Arabidopsis mutant deficient in 3 abscisic acid-activated protein kinases reveals critical roles in growth, reproduction, and stress. Proc. Natl. Acad. Sci. USA.

[47] Blum A. (2017). Osmotic adjustment is a prime drought stress adaptive engine in support of plant production: Osmotic adjustment and plant production. Plant Cell Environ..

[48] Fleta-Soriano E., Munné-Bosch S. (2016). Stress memory and the inevitable effects of drought: A physiological perspective. Front. Plant Sci..

[49] Lämke J., Brzezinka K., Bäurle I. (2016). HSFA2 Orchestrates transcriptional dynamics after heat stress in *Arabidopsis thaliana*. Transcription.

[50] Nechaev S., Adelman K. (2008). Promoter-proximal pol II: When stalling speeds things up. Cell Cycle.

[51] Sun R.-Z., Liu J., Wang Y.-Y., Deng X. (2021). DNA methylation-mediated modulation of rapid desiccation tolerance acquisition and dehydration stress memory in the resurrection plant *Boea hygrometrica*. PLoS Genet..

[52] Hake K., Romeis T. (2019). Protein kinase-mediated signalling in priming: Immune signal initiation, propagation, and establishment of long-term pathogen resistance in plants: Kinase-mediated signaling in defense priming. Plant Cell Environ..

[53] Stief A., Altmann S., Hoffmann K., Pant B.D., Scheible W.-R., Bäurle I. (2014). Arabidopsis MiR156 regulates tolerance to recurring environmental stress through SPL transcription factors. Plant Cell.

[54] Arshad M., Feyissa B.A., Amyot L., Aung B., Hannoufa A. (2017). MicroRNA156 improves drought stress tolerance in alfalfa (Medicago Sativa) by silencing SPL13. Plant Sci..

[55] Zheng R., Li H., Jiang R., Römheld V., Zhang F., Zhao F.-J. (2011). The role of root hairs in cadmium acquisition by barley. Environ. Pollut..

[56] Singroha G., Sharma P., Sunkur R. (2021). Current status of MicroRNA-mediated regulation of drought stress responses in cereals. Physiol. Plant.

[57] Candar-Cakir B., Arican E., Zhang B. (2016). Small RNA and degradome deep sequencing reveals drought-and tissue-specific micrornas and their important roles in drought-sensitive and drought-tolerant tomato genotypes. Plant Biotechnol. J..

[58] Baldwin I.T. (2006). Volatile signaling in plant-plant interactions: “Talking trees” in the genomics era. Science.

[59] Van Hulten M., Pelser M., van Loon L.C., Pieterse C.M.J., Ton J. (2006). Costs and benefits of priming for defense in arabidopsis. Proc. Natl. Acad. Sci. USA.

[60] Sedaghatmehr M., Thirumalaikumar V.P., Kamranfar I., Marmagne A., Masclaux-Daubresse C., Balazadeh S. (2019). A regulatory role of autophagy for resetting the memory of heat stress in plants: Role of autophagy in thermomemory. Plant Cell Environ..

[61] Couchoud M., Salon C., Girodet S., Jeudy C., Vernoud V., Prudent M. (2020). Pea efficiency of post-drought recovery relies on the strategy to fine-tune nitrogen nutrition. Front. Plant Sci..

[62] Jamil I.N., Remali J., Azizan K.A., Nor Muhammad N.A., Arita M., Goh H.-H., Aizat W.M. (2020). Systematic multi-omics integration (MOI) approach in plant systems biology. Front. Plant Sci..

[63] Lowder L.G., Zhang D., Baltes N.J., Paul J.W., Tang X., Zheng X., Voytas D.F., Hsieh T.-F., Zhang Y., Qi Y. (2015). A CRISPR/Cas9 toolbox for multiplexed plant genome editing and transcriptional regulation. Plant Physiol..

[64] Watson A., Ghosh S., Williams M.J., Cuddy W.S., Simmonds J., Rey M.-D., Asyraf Md Hatta M., Hinchliffe A., Steed A., Reynolds D. (2018). Speed breeding is a powerful tool to accelerate crop research and breeding. Nat. Plants.

[65] Meisner A., Jacquiod S., Snoek B.L., ten Hooven F.C., van der Putten W.H. (2018). Drought legacy effects on the composition of soil fungal and prokaryote communities. Front. Microbiol..

[66] Barnard R.L., Osborne C.A., Firestone M.K. (2013). Responses of soil bacterial and fungal communities to extreme desiccation and rewetting. ISME J..

[67] Birch H.F. (1958). The effect of soil drying on humus decomposition and nitrogen availability. Plant Soil.

[68] Barnard R.L., Blazewicz S.J., Firestone M.K. (2020). Rewetting of soil: Revisiting the origin of soil CO_2_ emissions. Soil Biol. Biochem..

[69] Bouskill N.J., Lim H.C., Borglin S., Salve R., Wood T.E., Silver W.L., Brodie E.L. (2013). Pre-exposure to drought increases the resistance of tropical forest soil bacterial communities to extended drought. ISME J..

[70] Preece C., Verbruggen E., Liu L., Weedon J.T., Peñuelas J. (2019). Effects of past and current drought on the composition and diversity of soil microbial communities. Soil Biol. Biochem..

[71] De Nijs E.A., Hicks L.C., Leizeaga A., Tietema A., Rousk J. (2019). Soil microbial moisture dependences and responses to drying–rewetting: The legacy of 18 years drought. Glob. Chang. Biol..

[72] Martiny J.B., Martiny A.C., Weihe C., Lu Y., Berlemont R., Brodie E.L., Goulden M.L., Treseder K.K., Allison S.D. (2017). Microbial legacies alter decomposition in response to simulated global change. ISME J..

[73] Averill C., Waring B.G., Hawkes C.V. (2016). Historical precipitation predictably alters the shape and magnitude of microbial functional response to soil moisture. Glob. Chang. Biol..

[74] Sardans J., Peñuelas J. (2005). Drought decreases soil enzyme activity in a mediterranean *Quercus Ilex* L. forest. Soil Biol. Biochem..

[75] Arnone J.A., Verburg P.S.J., Johnson D.W., Larsen J.D., Jasoni R.L., Lucchesi A.J., Batts C.M., von Nagy C., Coulombe W.G., Schorran D.E. (2008). Prolonged suppression of ecosystem carbon dioxide uptake after an anomalously warm year. Nature.

[76] Meisner A., de Deyn G.B., de Boer W., van der Putten W.H. (2013). Soil biotic legacy effects of extreme weather events influence plant invasiveness. Proc. Natl. Acad. Sci. USA.

[77] Marulanda A., Barea J.-M., Azcón R. (2009). Stimulation of plant growth and drought tolerance by native microorganisms (AM fungi and bacteria) from dry environments: Mechanisms related to bacterial effectiveness. J. Plant Growth Regul..

[78] Lau J.A., Lennon J.T. (2012). Rapid responses of soil microorganisms improve plant fitness in novel environments. Proc. Natl. Acad. Sci. USA.

[79] Kaisermann A., de Vries F.T., Griffiths R.I., Bardgett R.D. (2017). Legacy effects of drought on plant-soil feedbacks and plant-plant interactions. New Phytol..

[80] Preece C., Peñuelas J. (2016). Rhizodeposition under drought and consequences for soil communities and ecosystem resilience. Plant Soil.

[81] Hartman K., Tringe S.G. (2019). Interactions between plants and soil shaping the root microbiome under abiotic stress. Biochem. J..

[82] Bakker P.A.H.M., Pieterse C.M.J., de Jonge R., Berendsen R.L. (2018). The soil-borne legacy. Cell.

